# Correction: Evaluating outcomes of management targeting the recovery of a migratory songbird of conservation concern

**DOI:** 10.7717/peerj.4319/correction-1

**Published:** 2019-03-21

**Authors:** Henry M. Streby, Gunnar R. Kramer, Sean M. Peterson, David E. Andersen

**Affiliations:** 1 Department of Environmental Sciences, University of Toledo, Toledo, OH, United States of America; 2 Department of Environmental Science, Policy and Management, University of California, Berkeley, Berkeley, CA, United States of America; 3 Minnesota Cooperative Fish and Wildlife Research Unit, United States Geological Survey, St. Paul, MN, United States of America

Corrigendum:

After publication of the article “Evaluating outcomes of management targeting the recovery of a migratory songbird of conservation concern”, the American Bird Conservancy (ABC) and another reader raised concerns regarding the representation of the ABC’s involvement in the study site, representations of the site itself, the methodology used to map the territories, and the interpretation of the results. To address these concerns, the following corrections have been made by the authors, additional information has been included in the manuscript, and the “Expression of Concern” posted alongside this manuscript has now been removed.

There have been no substantive changes to the methods, results, conclusions, or inference drawn from the study.

The authors have included a supplemental pre-management document in which the planned management was described as “GWWA clearing” corroborating their original article and refuting alternative descriptions.

It is the opinion of the authors that the description of management in the revised manuscript now conforms to the latest description provided by the ABC and that the revisions of the management history did not influence the conclusions or inference of the original manuscript. The title now reads: “Evaluating outcomes of young forest management on a target species of conservation concern”.

The ABC’s involvement has been corrected to specify that brush management, or the shearing of shrubs and small trees, was prescribed by the ABC and its partners and was conducted by a contractor. Some references to a management initiative have been removed, and management practices are referred to as management prescriptions. Where appropriate, specific management practices, such as vegetation shearing, are named.

Descriptions of the ABC’s goals have been edited to specify that stated goal of vegetation management was to create shrubby young forest with high complexity that would benefit Golden-winged Warblers and other young-forest associated species within 4 years.

The main study site is now referred to as the treatment site (41 ha), and the control site remains the control site (30 ha).

Where appropriate, the target species has been specified as the Golden-winged Warbler.

The first period of observation of the treatment and control site, originally reported as 2011-2014 is now 2013- 2014. Although comparable observations were made on the treatment and control sites in 2011 and 2012, the authors agreed to remove those years because the 2011 and 2012 observations were made with a subset of the methods used in the remainder of the study, and excluding 2011 and 2012 made no difference in the analysis, results, or conclusions presented in the manuscript.

The coordinates of these sites have been changed to 46.528179° N, -93.407202° W to reflect the location of data collection and as opposed to the refuge headquarters.

It is now specified that while the ABC documentation describe brush management at the treatment site as a success, the authors’ models still predict a net loss of juvenile Golden-winged Warblers, and the estimates of those losses have not changed in the revised manuscript.

The Figure 2 legend has been updated to clarify that the photo in part B depicts vegetation during the first growing season after brush management intended to benefit the Golden-winged Warblers and associated forest species. The part C legend has also been updated to specify that Bakermans et al. (2015) and J. Larkin (an employee of the ABC) refer to the depicted landscape within the Bald Eagle State Park in Pennsylvania as being managed for Golden-winged Warblers.

*Corylus* spp. within the shrubland and young forest stands is now referred to as hazel.

In the “Management” section, management activity is now described as a single harvest prescription conducted across several areas across Rice Lake NWR. Of the approximately 40 ha of land that received management (i.e., vegetation shearing), 12 ha were located within the treatment site. Hydro-axing is now specified as a type of shearing.

A new paragraph has also been added to this section outlining post-management observations made by the ABC and the Rice Lake NWR regarding best management practices (BMPs) for Golden-winged Warblers in some areas and BMPs for the American Woodcock in others. The authors now specify that the areas they observed were identified prior to management as to receive Golden-winged Warbler BMPs, and the authors have added a pre-management document as a supplement confirming that the management was directed at Golden-winged Warblers.

The “Bird abundance monitoring” section is now the “Golden-winged Warbler abundance monitoring” section. In it, the authors outline methods that were used to conduct singing male surveys within the treatment and control sites: mist-netting, color banding males and females, and spot-mapping. The caution that spot-mapping is not adequate for delineating complete territories of Golden-winged Warblers, or for determining their mating success, which was not the objective of these methods, is now stated in this section (was previously only in the legend of Figure 3). Additional monitoring techniques are outlined, including behavioral observations, nest searches, netting, and individually marking females, that combined with spot-mapping, provide a means to identify core breeding areas of Golden-winged Warblers.

Figure 3 and its accompanying legend have also been corrected so that data from of each year (2013-2016) are presented. It is now specified that white polygons represent core breeding territories of Golden-winged Warblers. Figure 3 also now displays data from the control site for the same 4 years.

Recognition is now given that decisions at the local-refuge level can be influenced by a number of factors other than maximizing benefits for a single species of conservation concern.

In the Conclusions, the authors state that determining which species were targeted by brush management only after it has been completed may, in addition to not incorporating assessment efforts, obfuscate the effects and lead to incorrect conclusions about management outcomes.

They have also added that it is unclear in the Larkin et al. (2016) report whether areas identified as consistent with American Woodcock BMPs were included in the Larkin et al. (2016) assessment, despite being part of the same treatment.

Thanks are given in the Acknowledgements for comments made by readers on the original article.

“Survey data were collected as part of related studies of breeding Golden-winged Warbler ecology and migratory connectivity at the U.S. Geological Survey, Minnesota Cooperative Fish and Wildlife Research Unit” has been added to the end of the Acknowledgements.

Lastly, two references have been added to the reference list: Bakermans, Ziegler, & Larkin (2015) and Kramer et al. (2018).

Additionally, the title has been corrected for Buehler et al. (2007) so that *Verminora chrysoptera* now reads *Vermivora chrysoptera*, and proper nouns in the titles of Niemi et al. (2016), Streby et al. (2016), and Terhune II et al. (2016) have been copyedited.

Original PDF

