# Supplementary material for: Evaluating outcomes of young forest management on a target species of conservation concern
Source: PeerJ. 2018 Jan 29;6:e4319. doi: 10.7717/peerj.4319 (PMC5793708; doi:10.7717/peerj.4319)
Supplement: Supplemental Information 1 — Pre-management document from Rice Lake National Wildlife Refuge identifying the management to take place in our study area as “Sheared sites” and “GWWA Thinning”, confirming that Golden-winged Warbler was the primary pre-management target species of the habitat management we assessed. [file peerj-06-4319-s001.pdf]

UNITED STATES FISH & WILDLIFE SERVICE

ENVIRONMENTAL ACTION STATEMENT

Within the spirit and intent of the Council of Environmental Quality's regulations for implementing the National Environmental Policy Act (NEPA) and other statutes, orders, and policies that protect fish and wildlife resources, I have established the following administrative record and have determined that the action of (describe action):

Alder Shearing Project at Rice Lake NWR: Restore alder component on 225 acres by removing old decadent alder that provides minimal wildlife values by shearing or mowing, promoting new alder growth. Work will be done when ground is frozen to minimize soil disturbance.

__X__ is a categorical exclusion as provided by 516 DM 6, Appendix 1 and 516 DM 2, Appendix 1. No further documentation will therefore be made.

____ is found not to have significant environmental effects as determined by the attached Environmental Assessment and Finding of No Significant Impact.

____ is covered under an existing Environmental Assessment entitled ________________ as described in sub-section __________ which was approved on _______________

____ is found to have significant effects, and therefore further consideration of this action will require a notice of intent to be published in the Federal Register announcing the decision to prepare an EIS.

____ is not approved because of unacceptable environmental damage, or violation of Fish and Wildlife Service mandates, policy, regulations, or procedures.

____ is an emergency action within the context of 40 CFR 1506.11. Only those actions necessary to control the immediate impacts of the emergency will be taken. Other related actions remain subject to NEPA review.

Other supporting documents (list):

____ Environmental Assessment and FONSI

____ Public comments

____ Section 7 Form

____ Compatibility Determination

[signature]	3/1/2012	[signature]	3/6/12
(1) Initiator	Date	(2) RHPO * see attached RHPO form	Date
[signature]	3/6/12	Thomas O. Melius	3/6/12
(3) ARD	Date	(4) RD	Date

REQUEST FOR MIDWEST RHPO NHPA CLEARANCE

For Projects that have the Potential to Affect Historic Properties

Project Background:

Project Name: Young Forests for Minnesota Wildlife

Project Type: Alder Shearing Project

County/State: Aitkin County, MN On USFWS land? Yes

USFWS Program (NWR, WMD, NFH, PFW, FSH, ECS, MIB, NAWCA, Other (Name)): NWR

Project Location: Township 47 N , Range 23 W, Section(s): 18, 19, 30 & 31

Total Project Size (in Acres): 225 If road/trail, (linear ft, L and W): _________

USFWS Project Leader/Station: Walt Ford - Acting:R. Geboy/Rice Lake NWR Phone #: 218-768-2402

If there is another Federal/State/NGO partner, please name: Woodcock MN & 4 other MN USFWS NWR's.

Mandatory Attachments:

1.USGS Topographical map and aerial photo, ensuring that the project boundaries are exact.
- See attachments. (Topographic, Vegetative Cover, Refuge Boundary, Site Specific Maps)

2.Only Relevant Sections of Design Drawings (e.g. plan views)

- There are no design drawings; however the maps above identify the scope of proposed work. Aspects of 225 acres of brushy habitat will be sheared using hydro-axes, sickle-bar mowers, or similar pieces of equipment. Refuge staff will continue to monitor thr project site regularly until the project is completed. The method and timing of application has been established due to restrictions on soil disturbance and through consultation with other National Wildlife Refuge programs and through consultation with Woodcock Minnesota. All shearing activity must be completed November 1 – March 31.

3.History of ownership and environmental setting of the project area (add maps as necessary)

- History of ownership has been Rice Lake NWR since 1935. The environmental setting is a mixture of mature trees dominated by brushy areas. Hydric soils are the dominate soil type within the area of impact. Furthermore, historical landcover data indicates that the vegetative area consist of old field, hardwood/brushland, wet meadow, and shrub swamp habitat.

4.Details of anticipated project activities, such as ground disturbance (add maps as necessary)

- The activity will increase patchiness of homogenous hardwood/shrub stands and be managed on a rotational basis to benefit area wildlife. Specifically, the project will use heavy equipment (dozer w/shearing blade, tractor with mower, skid steer brush cutter) to clear trees/shrubs during frozen ground conditions, likely November 1 – March 31. Related ground disturbance will be minimized by completing activity during frozen conditions.

__Check here if there are known buildings/sites* in the project area (if none, stop here and sign).

*Sites are such places as artifact scatters, mounds or earthworks, cemeteries, privy pits, old foundations/ruins, bridges, historic roads/trails and trash pits/piles.

(see over)

Elements needed to be furnished to RHPO if there are buildings/sites in the project area:

1. Age of building(s)/site(s) or date(s) built: ________________ RPI #(s)__________________
2. Attach ground level photographs of both inside and outside of buildings/sites.
3. Attach aerial photo(s) showing buildings/sites (as close of detail as possible) or attach a sketch map illustrating the placement of the buildings/sites on the property, key the photos to the map.
4. Attach detailed descriptions of the buildings/sites with emphasis on their size, floor plans and architectural elements. Individually, what kind of shape are they in (good, fair or poor)?

Submitted by: __________________________ Date:______________ Phone #: _________________

RHPO Only **

Investigation	*Finding*	RHPO Project # ________________
__ No Field Survey Needed	X No Potential Effect. Justify: swampy areas to be wk in the winter	
__ Field Survey Done	__ No sites/buildings in APE. No Effect.	
__Phase I (ARPA? y / n)	__ Sites/Buildings present but none are Historic Properties. No Effect.	
__Phase II (ARPA? y / n)	__ Historic Properties present but there is No Effect/Adverse Effect.	
__Phase III (ARPA? y / n)	__ Historic Properties present, Resolved Adverse Effects (with MOA).	

__ Stipulations ____

[signature] 3/2/12

James E. Myster, USFWS Midwest RHPO Date

Rice Lake National Wildlife Refuge

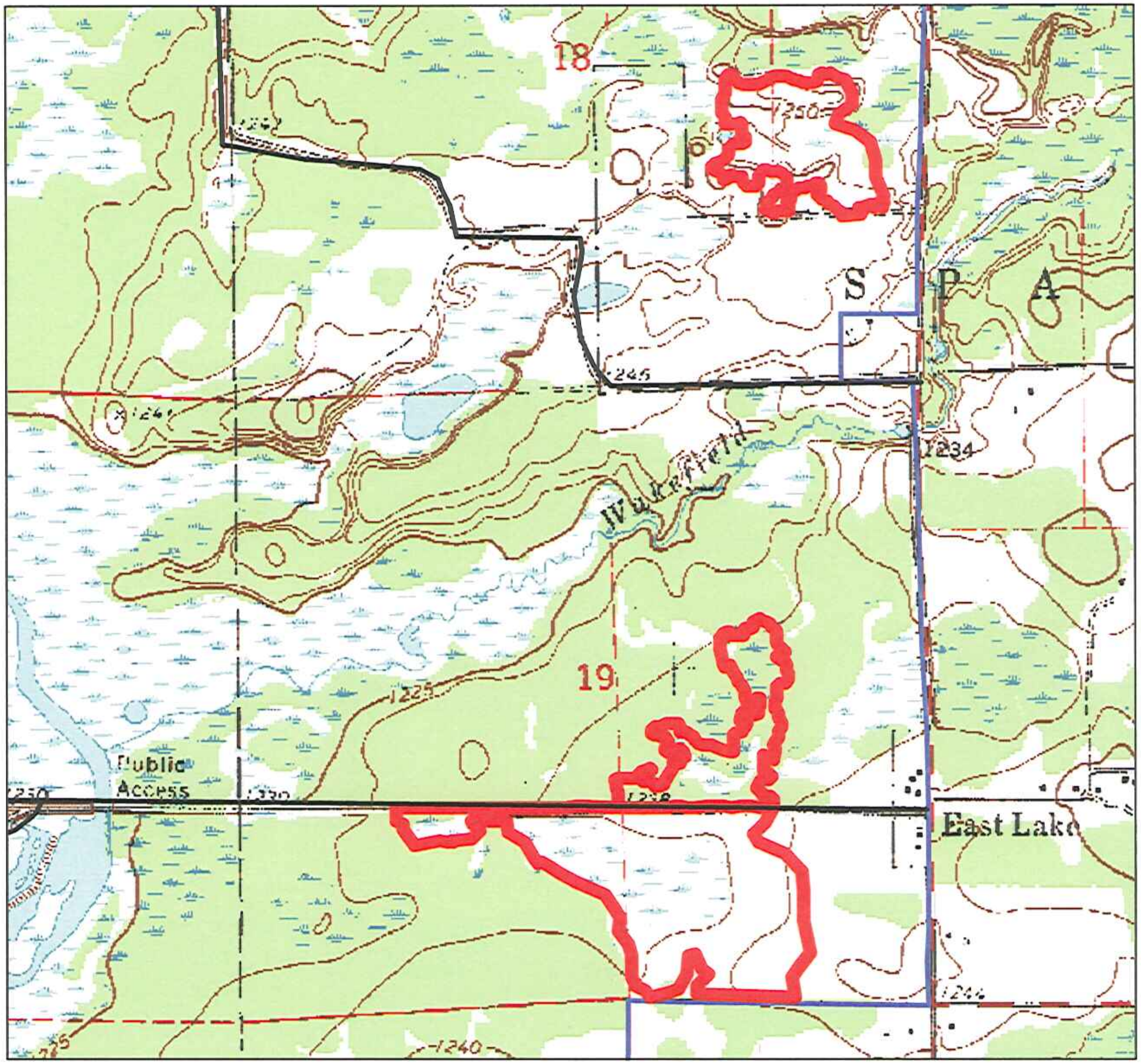

Legend

- Roads and trails
- Shearing Site
- Refuge boundary

0 0.5 1 Miles

Map Prepared by
Rich Geboy
2/10/12

Rice Lake National Wildlife Refuge

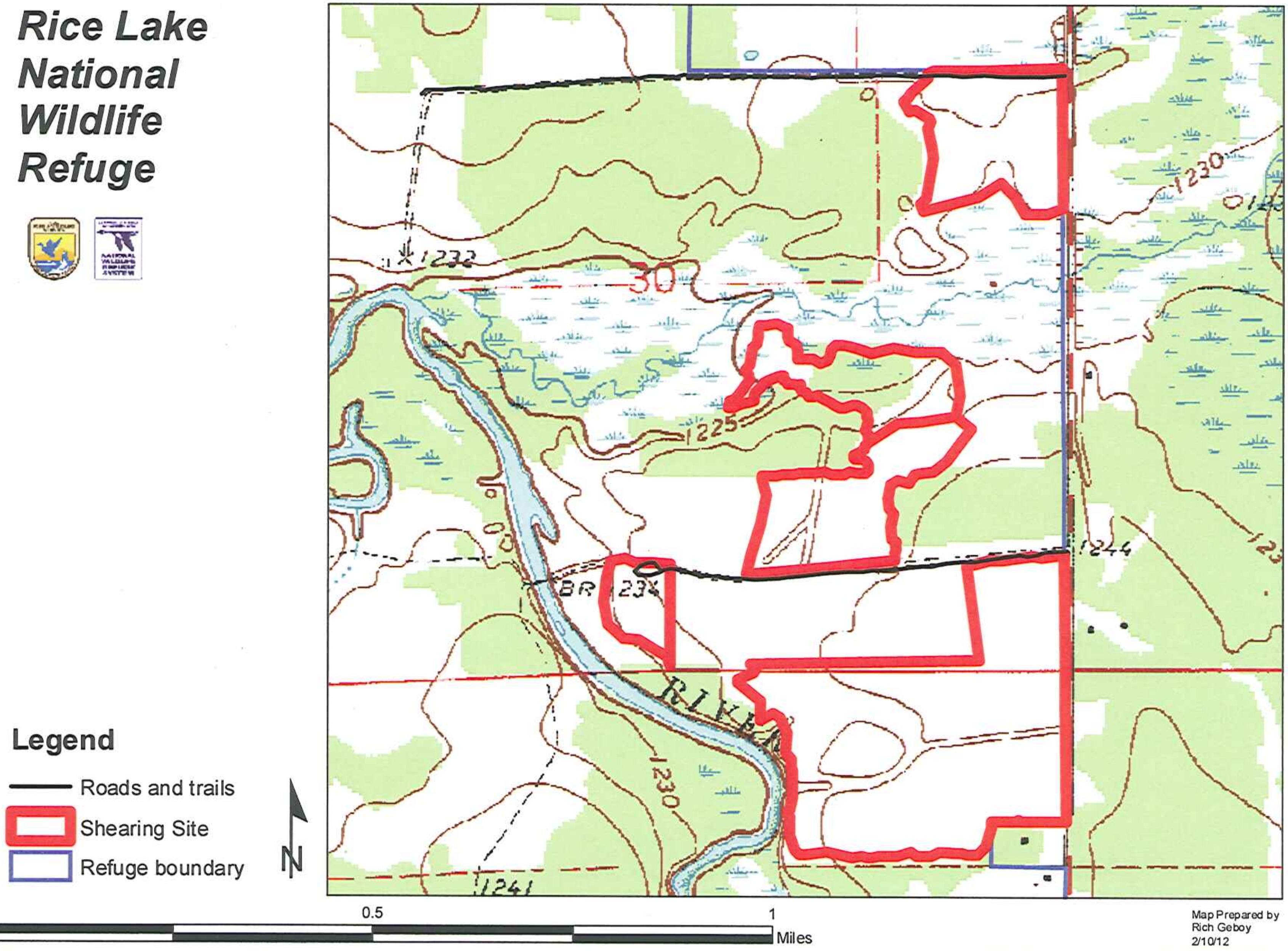

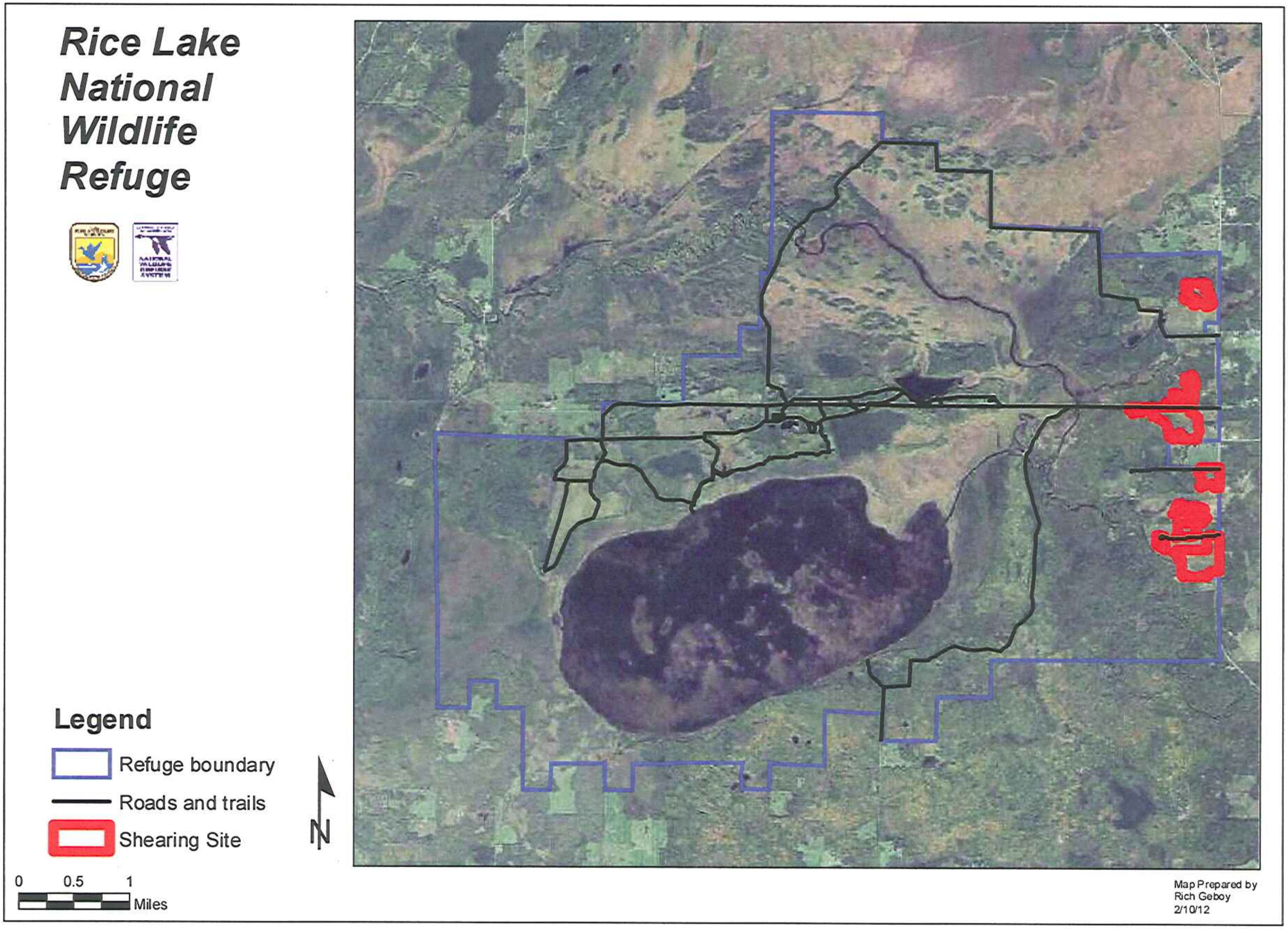
Rice Lake
National
Wildlife
Refuge
Legend
Refuge boundary
Roads and trails
Shearing Site
0
0.5
1
Miles
N
Map Prepared by
Rich Geboy
2/10/12

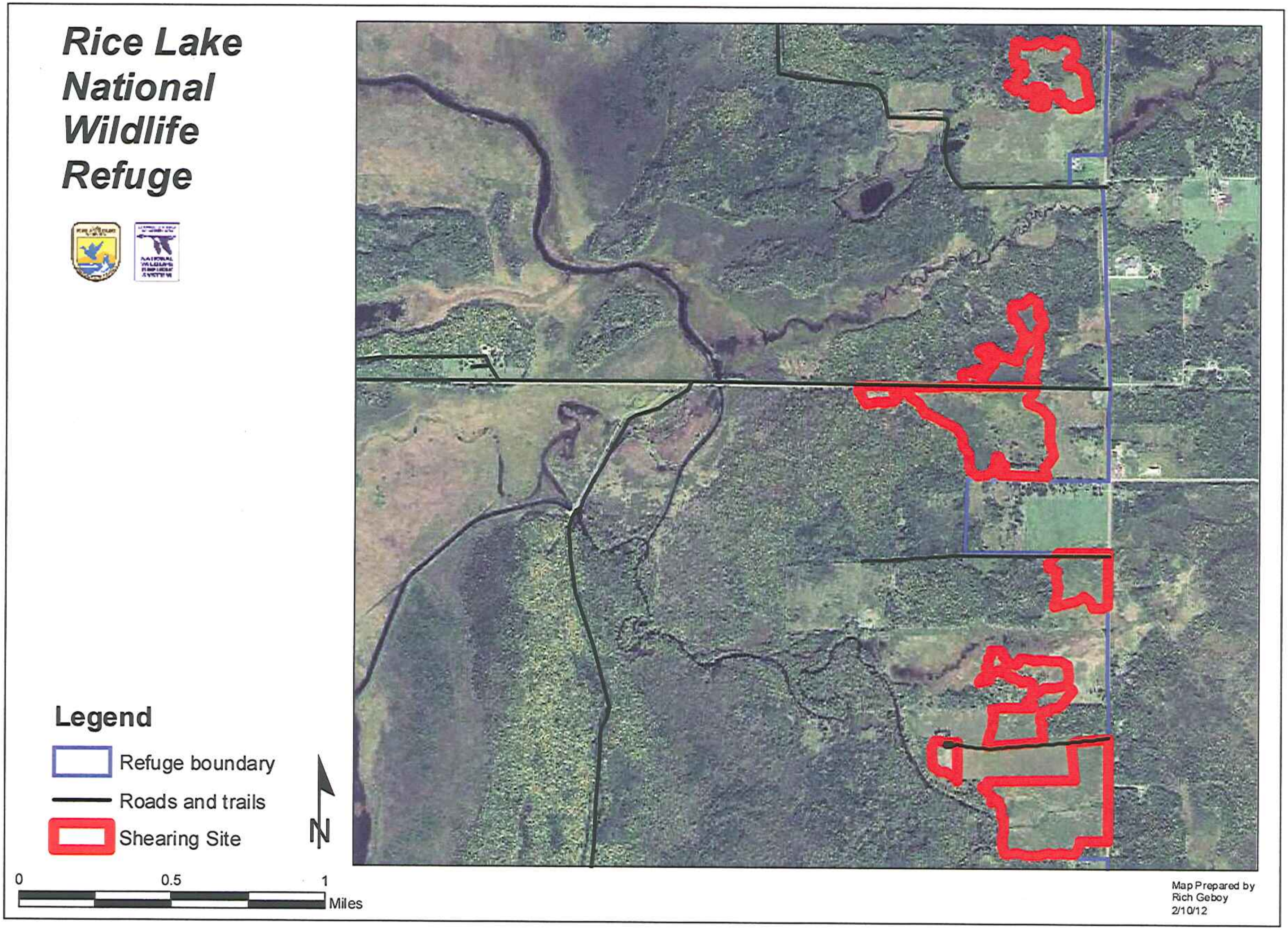
Rice Lake
National
Wildlife
Refuge
Legend
Refuge boundary
Roads and trails
Shearing Site
N
0
0.5
1
Miles
Map Prepared by
Rich Geboy
2/10/12
